# Development of Accelerated Test Method to Evaluate the Long-Term Thermal Performance of Fumed-Silica Vacuum Insulation Panels Using Accelerated Conditions

**DOI:** 10.3390/ma16196542

**Published:** 2023-10-03

**Authors:** Minjung Bae, Sunsook Kim, Jaesik Kang

**Affiliations:** 1 Department of Building Energy Research, Korea Institute of Civil Engineering and Building Technology, Goyang 10223, Republic of Korea; baeminjung@kict.re.kr; 2 Department of Smart Convergence Architecture, College of Engineering, Ajou University, Suwon 16499, Republic of Korea; kss@ajou.ac.kr

**Keywords:** vacuum insulation panel, accelerated test, long-term change

## Abstract

International standards for vacuum insulation panels (VIPs) include an accelerated test method and a minimum quality standard for evaluating their long-term thermal performance after 25 years. The accelerated test method consists of various tests according to the characteristics of the core material and requires six months (180 days) at minimum. Herein, we propose an accelerated method for determining the long-term thermal performance of fumed-silica VIPs by shortening the required time and simplifying the procedure. Highly accelerated conditions (80 °C and 70% Relative humidity (RH)) were set for the evaluation method, using the maximum temperature (80 °C) cited in international standards and compared with the accelerated test method under accelerated conditions (50 °C and 70% RH). The inner-pressure increase rate of the VIP samples after conditioning for approximately 70 days was similar to that after conditioning for 180 days under highly accelerated and accelerated conditions, respectively. In addition, the estimated long-term thermal conductivities of the fumed-silica VIP were derived as 0.0076 and 0.0054 W/m·K under highly accelerated and accelerated conditions, respectively. These accelerated methods can be used to produce fumed-silica VIPs with similar long-term thermal conductivities. Therefore, the accelerated test method for long-term thermal performance using the highly accelerated conditions can be evaluated using a test that involves conditioning the sample for approximately 70 days under 80 °C and 70% RH.

## 1. Introduction

As the demand for energy savings increases around the world, vacuum insulation panels (VIPs) are becoming attractive under strict standards, such as a concept with passive houses and zero-energy buildings. Among insulation materials with high potential in construction, VIPs are emerging as a new solution because they are thinner and more energy efficient than traditional insulation materials. When VIPs are applied to a building envelope, the excellent thermal performance can be achieved even with a small thickness, so the VIP is gaining popularity as a building insulation material to support the revitalization of zero-energy buildings and green remodeling projects. In particular, the indoor space can be maintained to be almost the same size before and after green remodeling construction.

The achievable thermal performance depends on the main components of the VIP: the core material, the barrier envelope, and getters/desiccants. Fumed silica (SiOx) is a well-known core material. Fumed silica has an appropriate pore structure and has low sensitivity to vacuum for VIPs. The fumed-silica VIP has an effective thermal conductivity of about 0.005 W/m·K measured [1,2,3]. The super-insulating material can secure thermal performance that is 5 to 10 times better than the conventional building insulation materials, such as expanded polystyrene, extruded polystyrene, polyurethane foam, and phenolic foam [4]. VIP is made by covering the core material with an envelope, extracting as much air as possible to create a vacuum, and then sealing the end of the envelope with heat welding. The envelope typically has a multi-layer structure consisting of a thin polymer film and aluminum foil. A polymer film is suitable for high-temperature bonding, and an aluminum foil or metallized thin film helps protect the inside of the VIP from atmospheric gases in a sealed envelope [5,6].

VIPs introduced as the high-performance insulation for the construction industry have been commercialized by several companies and are increasingly used in buildings in many countries [7]. For VIPs to be applied to buildings, the service life should be strictly guaranteed during the service life of a building (approximately 25 years).

First, the inside of VIP should be maintained at a constant vacuum to achieve excellent thermal conductivity stably for a long time [8]. However, over time, atmospheric gases penetrate the envelope, increasing the inside pressure of the VIP. The thermal conductivity of the VIP begins to increase, which reduces its thermal performance and reduces its service life [9]. The penetration of water vapor into the core material increases the thermal conductivity by increasing the inner pressure [10,11,12]. The time-dependent increase in thermal conductivity of the VIP depends on the increase in gas pressure and partial water vapor pressure in the core material [13]. The penetration of still air and water vapor through the envelope depends on the envelope composition and environmental condition [14]. The still air and water vapor transmittance of a VIP increases with temperature [15] and humidity [16]. And, changes in temperature and Relative humidity (RH) affect the transmittance [17,18]. Changes in the long-term thermal properties of VIPs have been documented in the literature. Theoretical predictions confirmed that barrier laminated aluminum foil can maintain its thermal properties for more than 100 years [19]. The service life of a VIP using a silica core and an envelope of aluminum foil can be 25 years, assuming a thermal conductivity of 0.0060 W/m·K on the core material. If a metalized polymeric envelope is used, the service life can be estimated at 25 years, assuming a thermal conductivity of 0.0080 W/m·K on the core material [13].

Baetens et al. [3] suggests an analytical model that describes the time-dependent changes in core conductivity by considering certain environmental conditions. A fumed-silica VIP of 50 cm × 50 cm × 1 cm starts with an initial thermal conductivity of 0.0040 W/m·K, and after 50 years, it becomes 0.0062 W/m·K for the laminated aluminum foil and 0.0099 W/m·K for the multilayer foil. Batard et al. [14] conducted simulations under constant boundary conditions and for several main materials. For example, at 20 °C, all VIPs experienced a change in thermal conductivity of approximately 0.002 W/m·K over 50 years. Another study [17] proposed a way to determine the long-term thermal performance of VIPs when they are installed in an actual building under variable external conditions. The silica VIP with an initial thermal conductivity of 0.004 W/m·K was analyzed to be 0.0055~0.0082 W/m·K for hydrophobicity and 0.0072~0.0095 W/m·K for hydrophilicity. Kim et al. [20] analyzed the long-term thermal performance of fumed-silica VIPs based on the nonlinear pressure–humidity dependence based on IEA/EBC Annex 39 [21]. After 25 years, the VIP was analyzed to be 0.0073 to 0.0082 W/m·K.

Under surrounding environmental conditions, the aging process of VIPs can last for decades, and its rate depends on the characteristics of VIPs. Pons et al. [16] conducted artificial aging (10 years) in the laboratory on panels with a fumed-silica core. The increase in weight and pressure of the VIP core was measured to be moderate at 30% RH and significant at 80% RH. The thermal conductivity increased by 0.0006 and 0.0022 W/m·K, respectively. Regarding the evaluation of VIP aging through field studies, Johansson et al. [22] monitored the temperature and relative humidity of remodeled building facades in Gothenburg for 5 years and found no signs of VIP deterioration. Installation of VIPs in the roof system by Molleti et al. [23] resulted in a change of approximately 10% in the initial thermal performance. Masi et al. [24] monitored a VIP-installed wall for 5 years and found that thermal conductivity increased by 10.6%.

As described above, considerable effort is required to verify the expected thermal conductivity degradation value when applied to buildings through long-term follow-up tests under surrounding environmental conditions. Using the VIP aging process in the laboratory to predict the thermal conductivity degradation value of VIPs is desirable for several reasons. There are several studies predicting the deteriorated thermal performance of VIPs over the service life of a building (approximately 25 years), but the different aging procedure conditions previously adopted make it difficult to compare study results. The manufacturer of VIPs should be able to present reliable initial thermal performance and long-term thermal performance analysis results to be applied to buildings. The study of accelerated test methods that derive deteriorated thermal performance through aging procedures in VIPs is still ongoing, and these methods are now beginning to be discussed.

Some international standards have already operated, including VIP accelerated testing methods. The European standard (EN 17140) and Japanese standard (JIS A 1488) [25,26] present accelerated test methods for long-term thermal performance evaluation of VIPs. The VIP accelerated test method being developed in the draft international standard (ISO/DIS) 16478 [27] consists of various tests that calculate the rate of change in thermal conductivity on the center of the panel over 25 years and should be evaluated for more than 6 months (about 180 days). If ISO/DIS 16478 is adopted as an international standard, the type of test and time required for one type of fumed-silica VIP product may be harsh. To use a VIP as the building insulation, the thermal performance needs to be evaluated quickly.

In this study, we propose a test method that shortens the required time to evaluate the long-term thermal performance of VIPs and simplifies the procedure. This study focuses on changes in the inner pressure and weight of VIP samples due to temperature and humidity conditions. The aging acceleration of fumed-silica VIPs is compared using the temperature and humidity conditions presented in ISO/DIS 16478 and conditions citable from international standards. Then, the expected thermal conductivity of fumed-silica VIPs after 25 years at 23 °C and 50% RH is determined, and the effectiveness of these results are analyzed.

## 2. Materials and Methods

### 2.1. Sample Preparation

**Figure 1:**
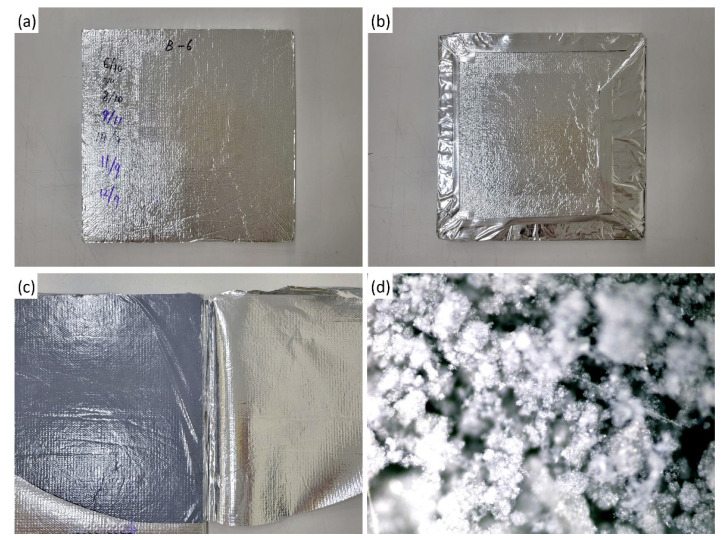
(**a**) Front of fumed-silica VIP sample, (**b**) Back of the sample, (**c**) Fumed-silica powder as a compression board, (**d**) Fumed-silica powder.

The fumed-silica VIP samples used in this study were obtained from K Manufacturer. As shown Figure 1, the core material was fumed-silica powder (SiOx), which was used as a compression board. Metalized composite films composed of nylon (15 μm), aluminum-coated polyethylene terephthalate (12 μm), aluminum-coated ethylene vinyl alcohol (12 μm), and linear low-density polyethylene (50 μm) were used as envelopes on both sides. The sample was 300 mm wide, 300 mm long, and 10 mm thick; however, a separately manufactured size was also used, according to the characteristics of the accessory test (e.g., the gas permeability of the envelope) required to perform the accelerated test method.

### 2.2. Methodology

Changes in the thermal performance of a VIP can be influenced by the characteristics of the core material, gases permeability of the envelope according to atmospheric pressure and water vapor pressure, and the size and thickness of the VIP. Because of the high pressure difference between the inside and outside of the VIP, atmospheric gas and water vapor slowly begin to penetrate the envelope [8]. Over a long period, as gas and water vapor in the atmosphere slowly permeate into the VIP, the gas pressure inside the VIP increases, and the thermal performance begins to deteriorate [9,13].

**Figure 2:**
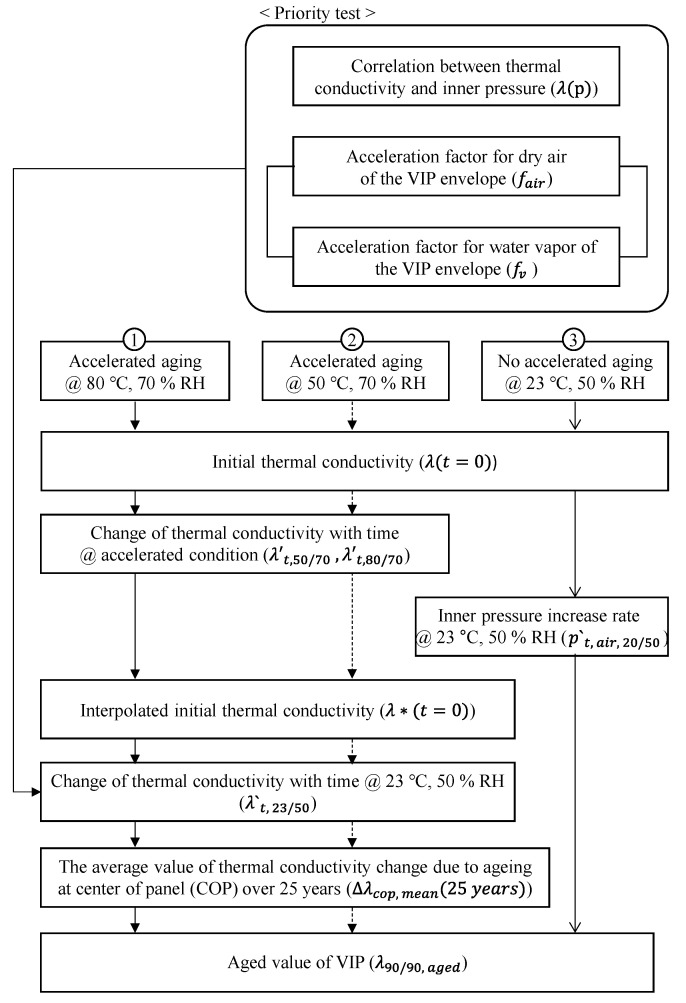
Flowchart of the research process for estimating the long-term thermal performance of fumed-silica VIP.

Under standard conditions (23 °C and 50% RH), the change in thermal conductivity of a VIP over time occurs very slowly. Tracking the thermal conductivity of the VIP over 25 years would require considerable effort. This study applied an accelerated test procedure to evaluate the long-term thermal performance of a VIP after 25 years, as shown in Figure 2. Before starting the accelerated test procedure, it is necessary to be able to describe the relationship between the inner pressure and thermal conductivity of the VIP as a function. Using the method given in Section 2.2.1, the thermal conductivity correlation with the inner pressure of the fumed-silica VIP sample was derived using the internal pressure regulator.

According to the Arrhenius Equation (1889), the higher the temperature, the more effective the collisions between the reacting molecules, resulting in a faster reaction rate [28]. The rate in all chemical reactions is highly dependent on temperature, and the amount of atmospheric gas permeation, such as dry air and water vapor, is affected by the difference between the internal and external area of VIPs. The accelerated test method utilizes high temperature and humidity (called ‘accelerated conditions’) to quickly induce changes in the thermal performance of VIPs. And, based on changes in thermal conductivity, weight, etc., it is scaled to the change in thermal performance after 25 years at 23 °C and 50% RH.

To scale the change in thermal performance promoted by accelerated conditions to 23 °C and 50% RH, the atmospheric gas permeation of the envelope is used as a key factor. In this study, the atmospheric gases that penetrate the barrier envelope and affect the long-term thermal performance of the VIP are limited to dry air and water vapor. The penetration performance of the envelope is calculated by the acceleration factors for dry air and water vapor of the envelope, as in Section 2.2.2. These acceleration factors are an indicator that indicates how much accelerated conditions can accelerate the change in the inner pressure of the VIP compared to standard conditions. In other words, the larger the value, the faster the change in the VIP’s inner pressure accelerates.

ISO/DIS 16478, which is under development, classifies VIP product types into silica and glass wool core materials and specifies different accelerated test methods. The acceleration conditions used in the test method for fumed-silica VIP are 50 °C and 70% RH, and the acceleration conditions for the glass wool VIP are four types: (1) 40 °C, 19% RH; (2) 50 °C, 70% RH; (3) 60 °C, 7% RH; and (4) 80 °C, 3% RH. This method is similar to JIS A 1488 and EN 17140, as well. However, EN 17140 establishes an accelerated test method to estimate the long-term thermal performance of a VIP using only the accelerated conditions of 50 °C and 70% RH, and the method of estimating the long-term thermal performance after 25 years under standard conditions is different, depending on whether the desiccant is applied.

ISO/DIS 16478 requires a variety of subtests to determine the thermal performance and quality of the fumed-silica VIP. As described in Section 2.2.3, the fumed-silica VIP needs to be conditioned at 50 °C and 70% RH for 6 months and measure the change in thermal conductivity. Ultimately, the time-dependent change rate of thermal conductivity needs to be found as a function. This function is used to convert the change rate of thermal conductivity that occurs over 25 years at 23 °C and 50% RH, and then to calculate the long-term thermal performance after 25 years, according to the method described in Section 2.2.4.

This study analyzed whether conditioning could be completed faster than the 6 months specified in the international standard when using the highest temperature and highest relative humidity conditions (80 °C and 70% RH) among the accelerated conditions used in the international test method. These conditions (80 °C and 70% RH) are defined as “highly accelerated conditions”. The accelerated test procedure for a fumed-silica VIP follows the methods specified in international standards, but evaluates VIP aging by considering two accelerated conditions. We plan to analyze whether using these two accelerated conditions can ensure similarity between long-term thermal performance estimates after 25 years.

ISO/DIS 16478 specifies the minimum standards required for VIPs, as shown in Table 1. The initial thermal conductivity at the center of the VIP should be less than 0.0050 W/m·K, and the long-term thermal conductivity change over time after 25 years should be less than 0.0100 W/m·K. In addition, the initial and long-term thermal resistance [m^2^·K/W] standards are also presented at the same time.

The thermal resistance was calculated using Equations (1) and (2), where $d_{N}$ is the thickness [mm]. For example, to satisfy the thermal resistance quality standard of a VIP with a thickness of 10 mm, the initial thermal conductivity must be 0.0063 W/m∙K or less, and the long-term thermal conductivity must be 0.013 W/m∙K or less.

(1) $$R_{cop,90/90}=\frac{d_{N}}{\lambda_{cop,90/90}}$$

(2) $$R_{cop,90/90,aged}=\frac{d_{N}}{\lambda_{cop,90/90,aged}}$$

We analyzed whether the estimated long-term thermal performance after 25 years could satisfy the minimum standards when an accelerated test was performed using accelerated and highly accelerated conditions. In addition, to examine the reliability of the two estimated values, the thermal conductivity of fumed-silica VIP was measured during follow-up tests for about 3 years under standard conditions. The time-dependent change rate of thermal conductivity is converted to the change rate in the inner pressure of the VIP to estimate the long-term thermal performance value after 25 years.

#### 2.2.1. Thermal Conductivity and Inner-Pressure Test of Fumed-Silica VIP

**Figure 3:**
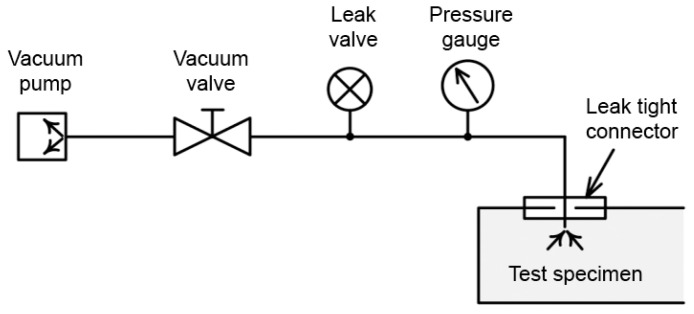
Scheme of the inner-pressure regulator of the VIP [Reprinted/adapted with permission from Ref. [27]. Copyright 2018, copyright ISO/TC 163/SC 03].

Tests for the thermal conductivity of VIPs were conducted to determine the correlation between the inner pressure and the thermal conductivity of fumed-silica VIPs. In accordance with KS L 9016 [29], the thermal conductivity was measured using a heat flow meter (HFM) manufactured by NETZSCH, while maintaining the inner pressure of the VIP step by step. As shown in Figure 3, a vacuum gauge (InstruTech CVM201, Longmont, CO, USA) capable of measuring the degree of the vacuum and a vacuum valve were attached to prepare the measurement sample. They were attached 15 cm from the HFM to avoid affecting thermal conductivity measurements.

Thermal conductivities were measured for the inner-pressure values of 10, 100, 1000, and 10,000 Pa, and these were interpolated using the least squares method. The relationship between the thermal conductivity and inner pressure of the VIP can be expressed by Equations (3) and (4); in these equations, $p_{1/2}$ is the value of the inner pressure of the VIP when the thermal conductivity of the VIP is half that of air (0.026 W/m·K).

The relationship between the thermal conductivity and internal pressure of the vacuum insulation material can be expressed using Equation (3); according to this equation, the inner pressure corresponding to the measured thermal conductivity can be converted, and the thermal conductivity corresponding to the measured internal pressure can be converted using Equation (4):

(3) $$\lambda \left(p\right)=\lambda_{0}+\frac{\lambda_{air}}{1+\frac{p_{1/2}}{p}}$$

(4) $$p=\frac{p_{1/2}}{\frac{\lambda_{air}}{\lambda \left(p\right)-\lambda_{0}}-1}$$

#### 2.2.2. Permeation Test on the VIP Envelope

The sample was 300 mm wide, 300 mm long, and 10 mm thick, considering the HFM. To measure the amount of dry air permeating the envelope, 16 g of calcium oxide (CaO) was used as an adsorbent for the six samples. To accelerate the amount of dry air and water vapor permeated through the envelope of the VIP, three samples were conditioned for 180 days under the accelerated conditions (50 °C and 70% RH), and the remaining three samples were also conditioned under standard conditions (23 °C and 50% RH).

The thermal conductivity of each sample was measured at 120, 150, and 180 days after the start of conditioning under both conditions. The measured thermal conductivities under accelerated and standard conditions were calculated as the average values of three samples. Then, the correlation between the internal pressure and thermal conductivity was established and converted into an internal pressure value corresponding to the thermal conductivity value, and the internal pressure change rate over 120–180 days was derived.

The inner-pressure increase rate for 120–180 days under standard conditions (${p^{\prime }}_{t,air,23/50}$) was calculated as the acceleration factor for dry air of the envelope ($f_{air}$) for the inner-pressure increase rate for the same period under accelerated conditions (${p^{\prime }}_{t,\;air,50/70}$), as shown in Equation (5).

(5) $$f_{air}=\frac{{p^{\prime }}_{t,air,50/70}}{{p^{\prime }}_{t,air,23/50}}$$

To measure the acceleration factor for the water vapor of the envelope ($f_{v}$), six samples with a width of 150 mm, length of 120 mm, and thickness of 7 mm were prepared. CaO (8 g) was used to absorb all the moisture that permeated through the envelope. Three samples were conditioned for 60 days under accelerated conditions of 50 °C and 70% RH, and the remaining three samples were conditioned under standard conditions of 23 °C and 50% RH for 60 days. Each sample was weighed every 10 days for 60 days, and the rate of weight increase during this period was assumed to be the moisture absorbed by the adsorbent.

The measured weights under accelerated and standard conditions were calculated as the average of three samples, and the weight value that increased over 30 days was divided by the surface area of the envelope for each sample to calculate the moisture permeability [g/(m^2^·day)]. $f_{v}$ was calculated as the water vapor permeability under standard conditions (${p^{\prime }}_{t,v,23/50}$) compared to that under accelerated conditions (${p^{\prime }}_{t,v,50/70}$). This ratio can be expressed using Equation (6):

(6) $$f_{v}=\frac{{p^{\prime }}_{t,v,50/70}}{{p^{\prime }}_{t,v,23/50}}$$

#### 2.2.3. Conditioning under Accelerated and Standard Conditions

To analyze the effect of the highly accelerated conditions of 80 °C and 70% RH on the inner-pressure increase rate of VIP, as mentioned in Section 2.2.2, the minimum conditioning period required under the highly accelerated conditions of 80 °C and 70% RH was calculated based on the dry air and water vapor permeation of the envelope.

Two samples with a width of 300 mm, length of 300 mm, and thickness of 10 mm were prepared, and their thermal conductivities were measured during conditioning under highly accelerated conditions. In addition, a sample was conditioned at 50 °C and 70% RH for 180 days, stored for approximately 1100 days (three years) under standard conditions, and compared with the inner-pressure increase rate of VIPs. Six samples of fumed-silica VIP with a width of 300 mm, length of 300 mm, and thickness of 10 mm were prepared. Three samples were conditioned under accelerated conditions (50 °C, 70% RH), and the remaining three samples were stored under standard conditions; the thermal conductivity of each sample was measured and calculated as an average value for each condition. The correlation between the thermal conductivity and inner pressure obtained through the test was converted to an inner-pressure value corresponding to the thermal conductivity.

#### 2.2.4. Estimating the Thermal Conductivity of VIP after 25 Years

The measured thermal conductivity while conditioning under two accelerated conditions (80 °C, 70% RH; 50 °C, 70% RH) can be derived as the change rate of thermal conductivity (${\lambda^{\prime }}_{t,80/70},\;{\lambda^{\prime }}_{t,50/70}$) over time using Equation (7).

(7) $${\lambda^{\prime }}_{t,80/70}(or\;{\lambda^{\prime }}_{t,50/70})=\frac{∆\lambda }{∆t}=\frac{{\lambda (t)}_{80/70}(or\;{\lambda (t)}_{50/70})-\lambda *(t=0)}{t}$$

The initial thermal conductivity estimated for *t* = 0 is defined as the corrected initial thermal conductivity ($\lambda *\left(t=0\right)$) and is conceptually different from the measured initial thermal conductivity. In addition, the thermal conductivity over time under the two accelerated conditions can be assumed, as shown in Equation (8).

(8) $${\lambda (t)}_{80/70}(or\;{\lambda (t)}_{50/70})=\lambda *\left(t=0\right)+{\lambda^{\prime }}_{t,80/70}(or\;{\lambda^{\prime }}_{t,50/70})\times t$$

Using the acceleration factor for dry air and water vapor of the envelope ($f_{v},\;f_{air}$), the change rate of thermal conductivity for VIPs under highly accelerated conditions can be switched with the change rate of thermal conductivity under the standard condition of 23 °C, 50% RH (${\lambda^{\prime }}_{t,23/50}$), as shown in Equation (9). ${\lambda^{\prime }}_{p}$, indicating the thermal conductivity increase rate due to the inner pressure, can be calculated by Equation (10).

(9) $${\lambda^{\prime }}_{t,23/50}=\frac{1}{f_{v}}\times {\lambda^{\prime }}_{t,80/70}(or\;{\lambda^{\prime }}_{t,50/70})+{\lambda^{\prime }}_{p}\times \frac{{p^{\prime }}_{t,air,23/50}}{d}\times (1-\frac{f_{air}}{f_{v}})$$

(10) $${\lambda^{\prime }}_{p}=\frac{\lambda_{air}}{p_{1/2}}$$

In addition, the thermal conductivity over time under the standard conditions of 23 °C, 50% RH can be assumed as follows.

(11) $${\lambda (t)}_{23/50}=\lambda *\left(t=0\right)+{\lambda^{\prime }}_{t,23/50}\times t$$

After 25 years, the thermal conductivity of the fumed-silica VIP was determined using Equation (12); the thermal conductivity after 25 years was determined by calculating the average rate of change ($∆\lambda_{cop,mean}(25\;years)$) for 25 years in the center of the VIP. The deviation between the interpolated initial thermal conductivity ($\lambda *\left(t=0\right)$) and the actual value ($\lambda \left(t=0\right)$) should be corrected because the change rate of thermal conductivity under accelerated conditions (${\lambda^{\prime }}_{t,80/70}$ or ${\lambda^{\prime }}_{t,50/70}$) does not reflect the initial thermal conductivity measured at the standard conditions of 23 °C and 50% RH. The rate of change in the thermal conductivity of the center of the VIP over 25 years can be expressed as a linear function over time, and the difference between the interpolated initial thermal conductivity ($\lambda *\left(t=0\right)$) and the actual value ($\lambda \left(t=0\right)$) is included as an intercept.

(12) $$\Delta \lambda_{cop,mean}\left(25\;years\right)=\lambda *\left(t=0\right)-\lambda \left(t=0\right)+{\lambda^{\prime }}_{t,23/50}\times \frac{25}{2}year$$

## 3. Results and Discussion

### 3.1. Correlation between Thermal Conductivity and Inner Pressure

Table 2 lists the measured thermal conductivities of the fumed-silica VIP with respect to the inner pressure. The thermal conductivity ($\lambda_{0}$) is 0.0034 W/m·K when the inner pressure is 10 Pa.

When the thermal conductivity of the fumed-silica VIP was 1/2 that of air, the inner-pressure value ($p_{1/2}$) was 58,343 Pa. Because an inner pressure below 59 Pa cannot be maintained owing to the characteristics of the core material, the correlation between the inner pressure and the thermal conductivity was calculated, and the thermal conductivity at 10 Pa was converted.

If the thermal conductivity of the fumed-silica VIP is the same size and components as that of the VIP sample used in this test, the corresponding inner-pressure value can be converted. Similarly, if the measured inner pressure is for the same VIP sample, it can be converted to thermal conductivity.

### 3.2. Permeation Test on the VIP Envelope

**Figure 4:**
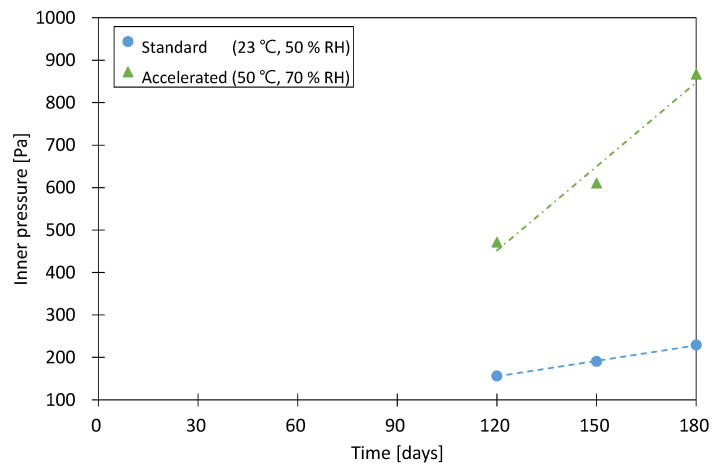
Inner pressure according to dry air permeation of the envelope.

Three samples were conditioned under the standard conditions and under the accelerated conditions, and the thermal conductivity was measured at 120, 150, and 180 days. The average thermal conductivities of the three samples were then calculated and converted to inner pressure, as shown in Figure 4.

According to experimental results, the inner-pressure increase rate under 23 °C and 50% RH (${p^{\prime }}_{t,air,23/50}$) was analyzed to be 1.21 Pa/day, and the inner-pressure increase rate under 50 °C and 70% RH (${p^{\prime }}_{t,air,50/70}$) was 6.60 Pa/day. The acceleration factor for the dry air of the envelope ($f_{air}$) was 5.4, which corresponded to a relatively high value compared to the acceleration factor ($f_{air}=4$) of the envelope (“metalized PET film”) presented in ISO/DIS 16478 [26].

**Figure 5:**
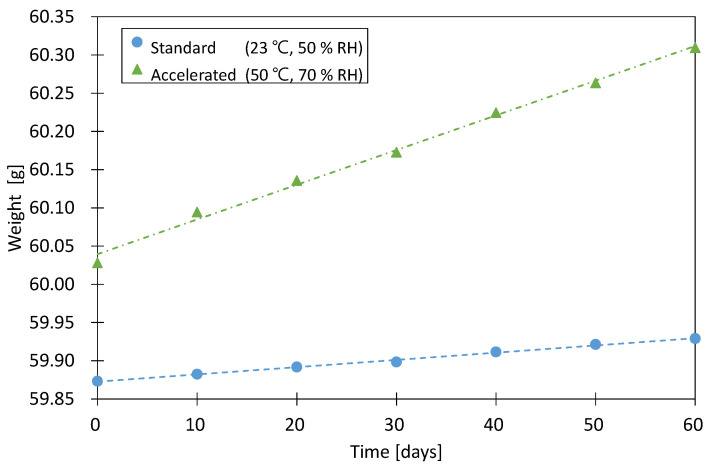
Weight according to water vapor permeation of the envelope.

Figure 5 shows the average weight of the three samples measured at intervals of 10 days from the start of conditioning. Based on the experiment results, the acceleration factor for the water vapor of the envelope ($f_{v}$) was 4.5, which corresponded to a relatively low value compared to the acceleration factor ($f_{v}=10$) of the envelope (“metalized PET film”) presented in ISO/DIS 16478 [26].

The acceleration factors for the dry air and water vapor of the envelope did not indicate perfectibility, implying that the permeation speed under the accelerated conditions was used relative to that under the standard condition. In other words, the higher the $f_{air}$ and $f_{v}$ values, the faster the rate of increase in the inner pressure of the envelope is.

### 3.3. Conditioning under Accelerated and Standard Conditions

#### 3.3.1. Determination of the Conditioning Period for Highly Accelerated Conditions

According to the Arrhenius Equation, as shown in Equation (13), the reaction rates of dry air and water vapor are affected by the temperature, and higher temperatures can promote faster reactions [28].

(13) $$\ln\;k=\ln A-\frac{E_{a}}{RT}$$

Therefore, assuming that the reaction rates of dry air and water vapor are affected only by temperature when the other conditions are the same, the acceleration factor for dry air in the envelope ($f_{air}$) can be defined as shown in Equation (14).

(14) $$k=f_{air}=exp\left[\frac{E_{a}}{R}\times \left(\frac{1}{T_{0}}-\frac{1}{T_{1}}\right)\right]$$

The acceleration factor for dry air in the envelope ($f_{air}$) used in this study was 5.4. Using this result and Equation (12), $E_{a}\cdot R^{-1}$, a constant composed of the activation energy ($E_{a}$) and gas constant (R), can be estimated as 5971.6. Under the highly accelerated conditions, the acceleration factor for dry air of the envelope ($f_{air}$) was 26.0, which could be calculated arithmetically. Accordingly, the result of conditioning for 180 days under accelerated conditions can match that for 37.4 days under the highly accelerated conditions. The acceleration factor for the water vapor of the envelope ($f_{v}$) can be defined as in Equation (15).

(15) $$k=f_{v}=\frac{p_{1}}{p_{0}}\times exp\left[\frac{E_{a}}{R}\times \left(\frac{1}{T_{0}}-\frac{1}{T_{1}}\right)\right]$$

The acceleration factor for the water vapor of the envelope ($f_{v}$) used in this study was 4.5. Under highly accelerated conditions, the acceleration factor for water vapor of the envelope ($f_{v}$) was 12.9, which could be calculated arithmetically. In other words, the results of conditioning for 180 days under accelerated conditions and those for 62.7 days under highly accelerated conditions can match.

Therefore, the conditioning period under the highly accelerated conditions was determined to be over 70 days to satisfy both 37.4 days calculated considering $f_{air}\;$ and 62.7 days calculated considering $f_{v}$. Under highly accelerated conditions, the test period could be shortened by about 40% of the required 180 days under accelerated conditions.

#### 3.3.2. Inner-Pressure Increase Rate under Accelerated and Standard Conditions

The highly accelerated conditions accelerated the permeation amount of dry air and water vapor through the envelope of the VIP more than the accelerated conditions, thereby rapidly accelerating the change in the thermal performance of the VIP. For the highly accelerated conditions set in this study to be useful, the inner-pressure increase rate of VIP conditioning for 180 days under the accelerated conditions and that of VIP conditioning for approximately 60–70 days at 80 °C and 70% RH should be similar.

Two samples were conditioned for 90 days, considering the minimum conditioning period required under the highly accelerated conditions. Thermal conductivity was measured at 10, 20, 30, 40, 70, and 90 days, including at the start of conditioning. In addition, thermal conductivity was measured at 0, 30, 60, 90, 120, and 180 days during conditioning for 180 days under accelerated conditions (50 °C, 70% RH). To analyze the validity of the two accelerated conditions, thermal conductivity was measured and stored for a long time under standard conditions. All the measured thermal conductivities were converted into inner pressures.

**Figure 6:**
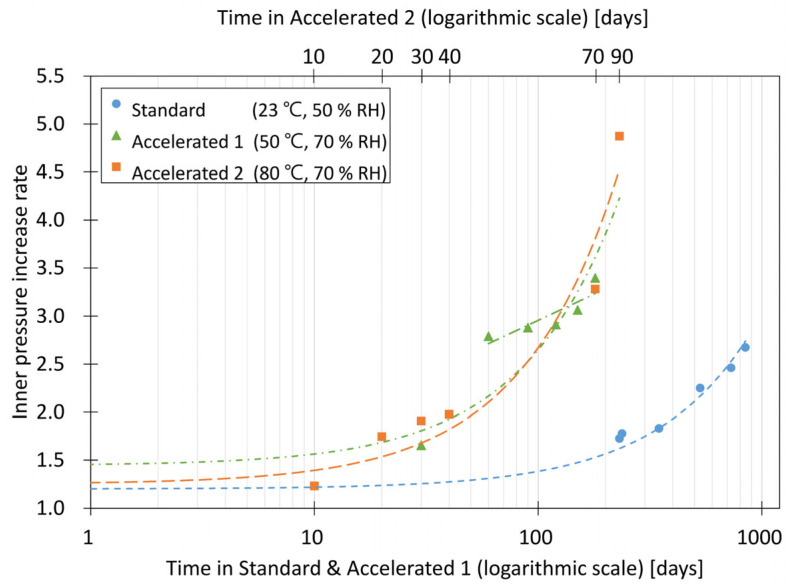
Inner-pressure increase rates of fumed-silica VIP under two accelerated conditions.

Figure 6 shows the log graph of the inner-pressure increase rate calculated under the standard condition, under the accelerated conditions, and under the highly accelerated conditions. The thermal conductivity was 0.0083 W/m·K at 90 days under the highly accelerated conditions, and the inner-pressure change rate of the VIP for 90 days was 4.87 Pa/day. The samples were exposed to highly accelerated conditions beyond the calculated conditioning period of 62.7 days and then exposed to a higher temperature than that under the accelerated conditions. This caused the inner-pressure increase rate to accelerate rapidly. Moreover, the inner-pressure increase rate of samples for 60–70 days after conditioning under 80 °C and 70% RH and that of samples for 60–180 days after conditioning under 50 °C and 70% RH are similar.

Meanwhile, the initial thermal conductivity of the VIP samples stored under the standard conditions of 23 °C and 50% RH was 0.0036 W/m·K, and after 3 years, the thermal performance gradually decreased to 0.0040 W/m·K. The long-term thermal conductivity changed by approximately 11% compared to the initial thermal conductivity. Moreover, the inner pressure of the VIP increased by approximately 2.7 times that after conditioning for 3 years under the standard conditions.

### 3.4. Estimating the Thermal Performance of VIP after 25 Years

Table 3 shows the variable values obtained from the experiments on the fumed-silica VIPs under the two accelerated conditions. Using these variables, the long-term thermal conductivity, which changes over time during the service life of a VIP (25 years), can be determined.

#### 3.4.1. Under Highly Accelerated Conditions

The initial thermal conductivity ($\lambda \left(t=0\right)$) of the VIP tested at 80 °C and 70% RH was 0.0047 W/m·K, whereas the interpolated initial thermal conductivity ($\lambda *\left(t=0\right)$) was 0.0062 W/m·K·s. The thermal conductivity increase rate with time (${\lambda^{\prime }}_{t,80/70}$) was 2.04 × 10^−10^ W/m·K·s, and the acceleration factor for the dry air and water vapor of the envelope calculated arithmetically in Section 3.3.1 were 26.0 and 12.9, respectively. Given these values, the thermal conductivity increase rate with time (${\lambda^{\prime }}_{t,23/50}$) under the standard conditions of 23 °C and 50% RH was derived as 3.49 × 10^−12^ W/m·K·s. Here, the thermal conductivity increase over 25 years ($∆\lambda_{cop,mean}(25\;years)$) in the center of the VIP was calculated as 0.0030 W/m·K, and the estimated value of thermal conductivity ($\lambda_{cop,90/90,aged}$) was 0.0076 W/m·K.

The long-term thermal performance of fumed-silica VIPs estimated using highly accelerated conditions satisfied the minimum quality standard (Table 1) of less than 0.0100 W/m·K, as specified in ISO/DIS 16478 under development. Considering the 10 mm thickness of the tested sample, when converted to thermal resistance using Equation (2), the value was 1.32 m^2^·K/W. This result also satisfies the minimum quality standard for long-term thermal resistance specified in ISO/DIS 16478, exceeding 0.8 m^2^·K/W. Therefore, the 10 mm thick fumed-silica VIP product in this study satisfied the long-term thermal performance quality standard.

#### 3.4.2. Under Accelerated Conditions

As shown in Table 2, the initial thermal conductivity ($\lambda \left(t=0\right)$) of the VIP tested at 50 °C and 70% RH was 0.0034 W/m·K, whereas the interpolated initial thermal conductivity ($\lambda *\left(t=0\right)$) was 0.0043 W/m·K·s. The thermal conductivity increase rate with time (${\lambda^{\prime }}_{t,50/70}$) was 1.90 × 10^−11^ W/m·K·s, and the acceleration factors for the dry air and water vapor of the envelope tested in Section 3.2 were 5.4 and 4.5, respectively. Using these values, the thermal conductivity increase rate with time (${\lambda^{\prime }}_{t,23/50}$) under the standard conditions of 23 °C and 50% RH was 2.9 × 10^−12^ W/m·K·s. In this case, the thermal conductivity increase over 25 years ($∆\lambda_{cop,mean}(25\;years)$) in the center of the VIP was calculated as 0.0021 W/m·K, and the estimated value of thermal conductivity ($\lambda_{cop,90/90,aged}$) was 0.0054 W/m·K.

The long-term thermal performance of the fumed-silica VIPs estimated using the accelerated conditions also satisfied the minimum quality standard (Table 1), which was less than 0.0100 W/m·K. Considering the 10 mm thickness of the tested sample, when converted using Equation (2), the thermal resistance was derived as 1.85 m^2^·K/W. This result also satisfied the minimum quality standard for long-term thermal resistance, exceeding 0.8 m^2^·K/W. These results are similar to the long-term thermal performance results (0.0076 W/m∙K, 1.32 m^2^∙K/W) estimated under the highly accelerated conditions of 80 °C and 70% RH.

#### 3.4.3. No Acceleration (under Standard Condition)

The inner-pressure increase rate (${p^{\prime }}_{t,air,23/50}$) of the fumed-silica VIP, which was monitored for three years under the standard condition, was 0.7925 Pa/day. Based on the converted value of the inner pressure of the sample collected over three years, the inner pressure over time can be expressed as a function, as shown in Equation (16). Using this equation, the inner pressure of the VIP sample after 25 years (9125 days) was derived as 7889.4 Pa.

(16) $$p\left(t\right)=0.7925\times 9125\;days+657.96$$

Using the correlation found in Section 3.1, the inner pressure can be converted into thermal conductivity. $\lambda_{air}$ is the thermal conductivity of dry air, 0.026 W/m ·K. Based on the measurement result for the 10 mm thick fumed-silica VIP sample, $\lambda_{0}$ was 0.0034 W/mλK, and $p_{1/2}$ was 58,343 Pa.

Consequently, the long-term thermal conductivity of the VIP sample was estimated to be 0.0065 W/m·K after 25 years under the standard condition. The value of 1.54 m^2^·K/W was calculated as a thermal resistance using Equation (2), considering the thickness of the sample. These results satisfied the minimum quality standard for the long-term thermal performance (less than 0.0100 W/m·K and greater than 0.8 m^2^·K/W). In addition, the results were similar to the long-term thermal performance (0.0076 W/m·K, 1.32 m^2^·K/W) estimated using the highly accelerated conditions.

#### 3.4.4. Comparing with the Estimated Thermal Performance of VIP after 25 Years

Table 4 shows the estimated long-term thermal performance of a fumed-silica VIP with 10 mm thickness under the highly accelerated conditions of 80 °C and 70% RH and under the accelerated conditions of 50 °C and 70% RH. In addition, the long-term thermal performance estimated by follow-up evaluation under the standard condition (23 °C, 50% RH), without accelerating the change in the thermal performance, is shown.

The long-term thermal performance of the fumed-silica VIP after 25 years, estimated through long-term follow-up testing under standard conditions, is 0.0040 W/m·K, and this value was 2.50 m^2^·K/W when it was converted to the thermal resistance. The minimum standards for a VIP (less than 0.0100 W/m·K, more than 0.8 m^2^·K/W) specified in ISO/DIS 16478 were satisfied, and the thermal performance was deteriorated about 11% compared to the initial thermal conductivity. However, when estimated by the acceleration test procedure using highly accelerated conditions, the long-term thermal performance was 0.0076 W/m·K, and the thermal performance was deteriorated about 162% compared to the initial thermal conductivity. Using acceleration conditions with the same method, the long-term thermal performance was 0.0054 W/m·K, and the thermal performance was deteriorated about 159% compared to the initial thermal conductivity.

The changes in the long-term thermal performance of VIPs published in the literature were evaluated through various methods, such as simulations, prediction models, and accelerated tests, as well as simple monitoring tests. Through these methods, the long-term thermal performance of fumed-silica VIPs is deteriorated by approximately 150-180% from the initial thermal conductivity [3,17,20], and the degradation of VIPs in this study are within the acceptable range.

Additionally, there was similarity between the two long-term thermal performances using the highly accelerated conditions of 80 °C and 70% RH and the accelerated conditions of 50 °C and 70% RH. In the accelerated test method, conditioning for 70 days using highly accelerated conditions can achieve similar results to conditioning for 180 days under accelerated conditions. Evaluating the long-term thermal performance of the VIP using the accelerated test method discussed in this study is only applicable to a fumed-silica VIP. Additionally, the following subtests should be conducted before the accelerated test:

- Relationship between thermal conductivity and the inner pressure;
- Acceleration factor for dry air and water vapor of the envelope.

The core material and barrier envelope used in these subtests are equal to the specimen conditioned using highly accelerated conditions. In particular, the width, length, and thickness of the specimen used to analyze the relationship between thermal conductivity and inner pressure should be the same as the fumed-silica VIP product to be evaluated by the accelerated test method.

Meanwhile, in order to increase the applicability of the accelerated test method of this study, it is necessary to analyze whether various fumed-silica VIP products could be evaluated using this method. In follow-up research, the accelerated test method will be applied to fumed-silica VIP products with various sizes and various types of envelopes and core materials.

## 4. Conclusions

The accelerated test method commonly utilized high temperature and humidity to quickly induce changes in the thermal performance of VIPs. The acceleration conditions used in the test method for the fumed-silica VIP were 50 °C and 70% RH, and fumed-silica VIPs needed to be conditioned for 180 days under these conditions. This study suggested a test method that shortens the required time to evaluate the long-term thermal performance of a VIP and simplifies the procedure comparing ISO/DIS 16478. The main conclusions drawn are as follows.

- According to the calculation, the results of conditioning for 180 days under 50 °C and 70% RH and those for 62.7 days under 80 °C and 70% RH would match. Based on the test results, the inner-pressure increase rate of samples for 60–70 days after conditioning under 80 °C and 70% RH and that of samples for 60–180 days after conditioning under 50 °C and 70% RH are similar.
- When estimated by the acceleration test procedure using highly accelerated conditions, the long-term thermal performance was 0.0076 W/m·K, and the thermal performance was deteriorated about 62% compared to the initial thermal conductivity. Using acceleration conditions with the same method, the long-term thermal performance was 0.0054 W/m·K, and the thermal performance was deteriorated about 59% compared to the initial thermal conductivity.
- These results satisfied the minimum standards for a VIP (less than 0.0100 W/m·K, more than 0.8 m^2^·K/W) specified in ISO/DIS 16478. Additionally, there was a similarity between the two long-term thermal performances using the highly accelerated conditions of 80 °C and 70% RH and the accelerated conditions of 50 °C and 70% RH. In the accelerated test method, conditioning for 70 days using highly accelerated conditions can achieve similar results to conditioning for 180 days under accelerated conditions.
- Evaluating the long-term thermal performance of a VIP using the accelerated test method discussed in this study is only applicable to a fumed-silica VIP. Additionally, the following subtests should be conducted before the accelerated test: relationship between thermal conductivity and the inner pressure; acceleration factor for dry air and water vapor of the envelope.
- The core material and barrier envelope used in these subtests are equal to the specimen conditioned using highly accelerated conditions. In particular, the width, length, and thickness of the specimen used to analyze the relationship between thermal conductivity and inner pressure should be the same as the fumed-silica VIP product to be evaluated by the accelerated test method.

## Figures and Tables

**Table 1:** The initial and long-term thermal performance of VIPs in ISO/DIS 16478.

Classification	Thermal Resistance [m^2^·K/W]	Thermal Conductivity [W/m·K]
Initial value of center of panel ($\lambda_{cop,90/90\;},R_{cop,90/90\;}$)	>1.6	<0.0050
Initial value including thermal bridging ($\lambda_{D}$)	-	Declare
Aged value of center of panel ($\lambda_{cop,90/90,aged\;},R_{cop,90/90,aged\;}$)	>0.8	<0.0100
Aged value including thermal bridging ($\lambda_{90/90,\;\;aged\;}$)	-	Declare

**Table 2:** The measured thermal conductivity according to the inner pressure of fumed-silica VIP.

Pressure [Pa]	Thermal Conductivity [W/m·K]
10	0.0034
59	0.0034
98.4	0.0035
960	0.0041
10,000	0.0095

**Table 3:** Variables for estimating long-term thermal performance of fumed-silica VIP over time.

Symbol	Unit	Accelerated Conditions	
		80 °C and 70% RH	50 °C and 70% RH
$\lambda_{0}$	W/m·K	0.0047	0.0034
$\lambda_{air}$	W/m·K	0.026	0.026
$p_{1/2}$	Pa	58,343	58,343
${\lambda^{\prime }}_{p}$	W/m·K·Pa	4.46 × 10^−7^	4.46 × 10^−7^
${\lambda^{\prime }}_{t,80/70}$	W/m·K·s	2.04 × 10^−10^	-
${\lambda^{\prime }}_{t,50/70}$	W/m·K·s	-	1.90 × 10^−11^
$\lambda *\left(t=0\right)$	W/m·K·s	0.0062	0.0043
$\lambda \left(t=0\right)$	W/m·K	0.0047	0.0034
$f_{air}$	-	26.0	5.4
$f_{v}$	-	12.9	4.5
${\lambda^{\prime }}_{t,23/50}$	W/m·K·s	3.49 × 10^−12^	2.90 × 10^−12^
Service life	years	25	25
$∆\lambda_{cop,mean}(25\;years)$	W/m·K	0.0030	0.0021
$\lambda_{cop,90/90,aged}$	W/m·K	0.0076	0.0054

**Table 4:** Initial and long-term thermal performance of fumed-silica VIPs.

Criteria		Thermal Conductivity [W/m·K]		Thermal Resistance [m^2^·K/W]	
		Initial	Aged	Initial	Aged
		<0.0050	<0.0100	>1.6	>0.8
Test condition	Highly accelerated	0.0047	0.0076	2.13	1.32
	Accelerated	0.0034	0.0054	2.94	1.85
	Not accelerated	0.0036	0.0040	2.78	2.50

## Data Availability

Not applicable.
